# Kidney care at NICU discharge and follow-up recommendations for preterm infants<34 weeks

**DOI:** 10.1038/s41372-026-02597-x

**Published:** 2026-02-26

**Authors:** Cara L. Slagle, Jennifer L. Chmielewski, Jennifer A. Rumpel, Keia R. Sanderson, Meredith P. Schuh, Michelle C. Starr, Jeffrey L. Segar, Namasivayam Ambalavanan, David Askenazi, David Selewski, Jennifer R. Charlton, Matthew W. Harer

**Affiliations:** 1 Division of Neonatal-Perinatal Medicine, Department of Pediatrics, Indiana University School of Medicine, Indianapolis, IN USA; 2 Division of Neonatology, Department of Pediatrics, University of Arkansas for Medical Sciences, Little Rock, AR USA; 3 Department of Pediatrics, University of North Carolina, Chapel Hill, NC USA; 4 Division of Nephrology and Hypertension, Cincinnati Children’s Hospital Medical Center, Department of Pediatrics, University of Cincinnati College of Medicine, Cincinnati, USA; 5 Division of Pediatric Nephrology, Department of Pediatrics, Indiana University School of Medicine, Indianapolis, IN USA; 6 Division of Child Health Service Research, Department of Pediatrics, Indiana University School of Medicine, Indianapolis, IN USA; 7 Division of Neonatology, Department of Pediatrics, Medical College of Wisconsin, Milwaukee, WI USA; 8 Division of Neonatology, Department of Pediatrics, University of Alabama at Birmingham, Birmingham, AL USA; 9 Division of Nephrology, Department of Pediatrics, University of Alabama at Birmingham, Birmingham, AL USA; 10 Division of Nephrology, Department of Pediatrics, University of Arkansas for Medical Sciences, Little Rock, AR USA; 11 Division of Pediatric Nephrology, Department of Pediatrics, University of Virginia School of Medicine, Charlottesville, VA USA; 12 Division of Neonatology, Department of Pediatrics, University of Wisconsin School of Medicine and Public Health, Madison, WI USA; 13 Division of Pediatric Nephrology, Department of Pediatrics, University of Miami/Holtz Children’s Hospital, Miami, FL USA; 14 Center for Research, Education, Quality, and Safety Pediatrix Medical Group, Sunrise, FL USA; 15 University of Pittsburgh School of Medicine, UPMC Pittsburgh Children’s Hospital, Pittsburgh, PA USA; 16 Division of Pediatric Nephrology and Hypertension, Department of Pediatrics, Nationwide Children’s Hospital/The Ohio State University College of Medicine, Columbus, OH USA; 17 Division of Neonatology, Department of Pediatrics, Indiana University School of Medicine, Indianapolis, IN USA; 18 Division of Nephrology, Department of Pediatrics, Indiana University School of Medicine, Indianapolis, IN USA; 19 Division of Neonatal-Perinatal Medicine, Department of Pediatrics, Emory University School of Medicine, Atlanta, GA USA; 20 Department of Pediatrics, University of Colorado School of Medicine, Anschutz medical Campus, Aurora, CO USA; 21 University of Michigan School of Medicine, C.S. Mott Children’s Hospital, Ann Arbor, MI USA; 22 Division of Neonatology, Department of Pediatrics, Golisano Children’s Hospital University of Rochester, Rochester, NY USA; 23 Division of Neonatology, Department of Pediatrics, University of Kentucky, Lexington, KY USA; 24 Division of Pediatric Nephrology, Department of Pediatrics, Montefiore Medical Center, Bronx, NY USA; 25 Division of Pediatric Nephrology, Department of Pediatrics, University of Washington/Seattle Children’s Hospital, Seattle, WA USA; 26 Division of Pediatric Nephrology, Department of Pediatrics, Medical College of Wisconsin/Children’s Wisconsin, Milwaukee, WI USA; 27 Division of Pediatric Nephrology, Department of Pediatrics, Texas Children’s Hospital, Houston, TX USA; 28 Division of Pediatric Cardiology, Department of Pediatrics, Nationwide Children’s Hospital/The Ohio State University College of Medicine, Columbus, OH USA; 29 Department of Pediatrics, Lucile Packard Children’s Hospital Stanford, Palo Alto, California, Palo Alto, CA USA; 30 Division of Nephrology, Department of Medicine, Indiana University School of Medicine, Indianapolis, IN USA; 31 Division of Pediatric Nephrology, Department of Pediatrics, Stanford University, Palo Alto, CA USA; 32 Division of Nephrology, Dialysis and Transplantation, Stead Family Department of Pediatrics, The University of Iowa, Iowa City, IA USA; 33 Division of Neonatology, Department of Pediatrics, Texas Children’s Hospital, Houston, TX USA; 34 Perinatal Institute, Department of Neonatology, Department of Pediatrics, Cincinnati Children’s Hospital Medical Center, Cincinnati, OH USA; 35 Division of Pediatric Nephrology, Department of Pediatrics, Ann & Robert H. Lurie Children’s Hospital of Chicago, Chicago, IL USA; 36 Division of Neonatology and General Pediatrics, Department of Pediatrics, Indiana University School of Medicine, Indianapolis, IN USA; 37 Children’s of Alabama, Division of Pediatric Nephrology, University of Alabama at Birmingham, Birmingham, AL USA; 38 Division of Neonatal-Perinatal Medicine, Department of Pediatrics, Medical University of South Carolina, Charleston, SC USA; 39 Division of Perinatal-Neonatal Medicine, Department of Pediatrics, University of Alabama at Birmingham, Birmingham, AL USA; 40 Division of Neonatal-Perinatal Medicine, Department of Pediatrics, University of British Columbia, Vancouver, BC Canada

**Keywords:** Paediatric kidney disease, Chronic kidney disease

## Abstract

Preterm birth increases the risk of chronic kidney disease (CKD) and hypertension later in life. To address these risks, the National Institutes of Health sponsored the Neonatal Kidney Health Consensus Workshop in February 2024, where a multidisciplinary group of experts reviewed current evidence, identified knowledge gaps, and developed consensus-based recommendations for kidney health follow-up in infants born <34 weeks. Key recommendations include a kidney evaluation before NICU discharge and at two years of age, with comprehensive kidney assessment for those at highest risk (birth <28 weeks, with acute kidney injury, intrauterine growth restriction, or small for gestational age). Cohesive, evidence-based parental education at multiple timepoints was emphasized to support early CKD detection and long-term management. This consensus provides a framework to optimize follow up and highlights research priorities aimed at improving risk stratification, early diagnosis, and interventions in individuals born preterm.

## Introduction

Although survival of preterm infants has dramatically improved, these children remain vulnerable to long-term complications [1–3], and kidney outcomes are now increasingly recognized as a critical concern [4–9]. As CKD is often silent, identification may be delayed until disease is advanced [10, 11]. While there are no comprehensive guidelines to evaluate for CKD and hypertension following a history of preterm birth, the American Academy of Pediatrics (AAP) recommended blood pressure (BP) monitoring for a variety of higher risk neonatal populations [12]. Additionally, Kidney Diseases Improving Global Outcomes (KDIGO) recognized preterm birth as a CKD risk factor [13]. Despite these recommendations, guidelines for the long-term monitoring of kidney disease in preterm neonates are lacking [10, 11, 14].

The first Neonatal Kidney Health Consensus workshop was convened in Indianapolis, Indiana, on February 27-28, 2024, to begin to address these pressing issues [15]. The preterm workgroup sought to address three questions on preterm birth and kidney health:

(1) What is the effect of preterm birth on kidney health over the life course?

(2) What is the effect of AKI in preterm infants on kidney health over the life course?

(3) What is the effect of other modifying factors in preterm infants on kidney health over the life course?

This manuscript details the work performed and conclusions drawn by the workgroup.

## Methods

The preterm work group, one of three workgroups of the NIH-supported “Consensus Workshop to Address Kidney Health in NICU Graduates” was composed of a diverse group of experts in neonatology and nephrology from the United States [15]. Using current literature supplemented by expert opinion, this workgroup sought to (1) develop recommendations for preterm kidney health follow-up, (2) define gaps in knowledge, and (3) establish research priorities.

Pre-workshop activities included a comprehensive literature search performed with Covidence software (Supplement 1). Articles were screened for inclusion and full review (JRC and MWH). Each reviewer documented strength of evidence and key points from the assigned study.

The preterm workgroup used a modified Delphi method to review findings, develop recommendations, and identify research gaps and priorities [16, 17]. During the workshop, recommendations were presented to all in attendance, which included critical care, neonatology, cardiology, and nephrology specialists in addition to a primary care provider, an expert in equity and care delivery and a family representative [15]. Feedback was incorporated into final revisions before a formalized vote with 2/3 support required for adoption. SQUIRE guidelines were used to identify key elements for inclusion in the manuscript, though the project itself was not conducted as a formal quality improvement initiative [18].

## Results

### Why should preterm neonates have kidney health monitoring?

The impact of perinatal factors and preterm birth on kidney health over the life course has become increasingly clear [17]. Studies consistently indicate that preterm birth is a risk factor for the future development of CKD and hypertension (HTN) [5–7, 19–21]. Preterm infants experience disrupted nephrogenesis which typically does not conclude until 34–36 weeks GA [22, 23]. Following delivery, preterm infants are exposed to extrauterine environmental stressors, including high or low levels of oxygen, hypotension, inadequate nutrition, and nephrotoxic medications, that may further disrupt postnatal nephron formation [24–30].

Emerging preclinical studies provide mechanistic insights into the development and progression of CKD [31, 32]. Maternal co-morbidities such as gestational diabetes and hypertensive disorders, along with nutritional deficiencies during gestation, including low levels of vitamin A, protein, iron, zinc, and sodium, have been shown to have an effect on nephrogenesis and reduce nephron numbers in offspring [33]. Studies in preterm baboons raised in conditions resembling NICUs confirm disruptions in nephrogenesis [31, 32]. The long-term consequences of preterm birth on kidney health have been explored in other animal models, such as in rats and mice, in which nephrogenesis normally occurs postnatally. Single-nephron hyperfiltration increases GFR in context of nephron loss. This contributes to further nephron loss and subsequent CKD and HTN [4, 7].

### Who should have kidney health monitoring after NICU discharge?

**Fig. 1:**
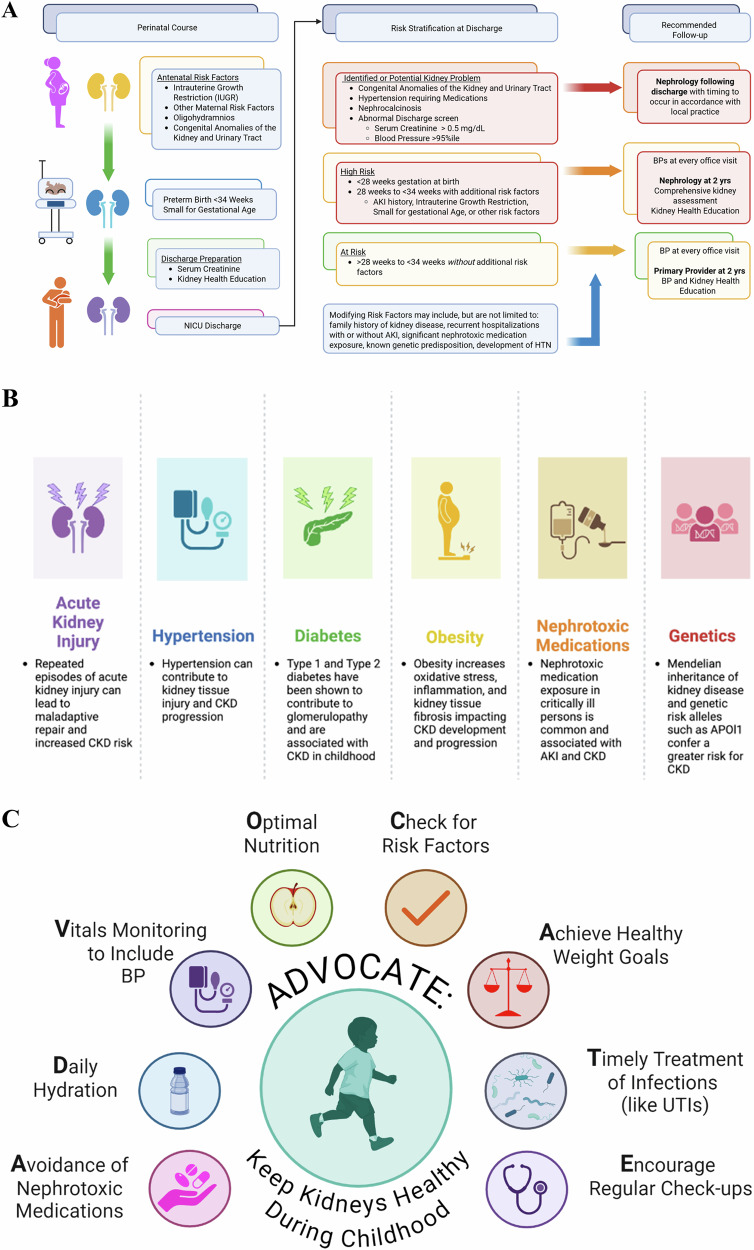
Consensus recommendations, risk modifiers, and education. **A** Consensus recommendations and Risk Stratification for outpatient kidney health follow-up for infants at risk of kidney disease during childhood. **B** Risk Modifiers for Kidney Disease Progression during childhood and adulthood. **C** Education related to Keeping Kidneys Healthy During Childhood.

We define infants *at-risk* of CKD if they were <34 weeks GA. We defined infants at *high-risk* of CKD if they were <28 weeks GA, birthweight <1500 g, or were at-risk and had an additional risk factor (Table 1: Recommendations, Fig. 1A).

**Table 1:** Consensus recommendations for outpatient kidney health follow-up for preterm infants.

Preterm infants (<34 week) recommendations		Level of Evidence
*Overall Recommendations*		
*1*	Preterm infants (born <34 weeks’ gestation) should have a kidney health evaluation prior to NICU discharge that includes a properly obtained blood pressure (BP), and serum creatinine measurement, with parent(s)/guardian(s) provided kidney health education and resources. Infants identified with evidence of kidney disease at discharge (one or more of the following: serum creatinine >0.5 mg/dL, BP >95th percentile for age, hypertension treated with medications, nephrocalcinosis, or CAKUT), should receive follow-up with pediatric nephrology according to local guidance.	3
2	*High-risk* preterm infants ( < 28 weeks’ gestation, birthweight <1500 grams, or history of AKI or dialysis) should have a comprehensive kidney health assessment *(BP, serum creatinine & Cystatin C, urine protein and/or urine microalbumin to urine creatinine ratio)* in conjunction with standard care at two years of age, or sooner if there are significant exposures or events that modulate risk	3
3	*At-risk* preterm infants (28 to <34 weeks’ gestation) should have BP measurement and kidney health education at two years of age in conjunction with standard care. Providers should consider comprehensive kidney health assessment if there are significant exposures or events that modulate risk, including history of AKI and/or perinatal growth restriction	3
***Who should have kidney health monitoring?***		
*Extremely Preterm Neonates (<28 wks)*	*We recommend that in addition to kidney health education and BP assessment at every healthcare encounter, infants born* < *28 weeks GA have a two-year (either chronologic or corrected age) comprehensive kidney health assessment (BP, serum creatinine & Cystatin C, urine protein and/or urine microalbumin to urine creatinine ratio). Assessment may occur sooner if additional CKD risk factors or significant exposures modulating CKD risk are present*	3
*Preterm Neonates 28 to* < *34 wks*	*We recommend infants born 28–34 weeks GA have kidney health education and a BP assessment at two years of age in line with current AAP guidelines*	5
*Modifying Risk Factors:*		
*AKI*	*We recommend that AKI is defined using modified neonatal KDIGO criteria, with attention paid to accurate diagnosis, documentation, and communication at the time of discharge* [25, 65, 66]. *Further, we note that preterm infants (28–34 weeks’ gestation) with AKI are at high risk for CKD and recommend that they should undergo comprehensive kidney health assessment at two years of age (serum creatinine, urine protein) in addition to BP assessment and kidney education*.	3
*IUGR/SGA*	*We recommend that preterm infants (28–34 weeks’ gestation) born* < *1500 g (*<10th percentile for weight at 33 weeks’ GA and <3rd percentile at 34 weeks’ GA) *are at high risk for CKD and should undergo comprehensive kidney health assessment (BP, serum creatinine & Cystatin C, urine protein and/or urine microalbumin to urine creatinine ratio) at two years of age in addition to BP assessment and kidney education*	3
*Other*	*We acknowledge that family history, rehospitalization, and other risk factors should be taken into consideration when determining risk assessment for follow-up of preterm infants*	4
***What tests are indicated?***		
*Serum*	*We recommend that serum cystatin C and serum creatinine should be obtained at two years of age for those* < *28 weeks GA or in those 29 to* < *34 weeks GA with modifying risk factors or concerns. Serum cystatin C and serum creatinine may be obtained at a two-year visit by the general provider optionally irrespective of risk factors*.	3
*Urine*	*We recommend that those at high-risk (preterm infants* < *28 weeks GA or those born 28 to* < *34 weeks GA but with* ≥ *1 modifying risk factor) have urine albumin to creatinine ratios at a two-year follow-up visit. For infants 28 weeks GA to* < *34 weeks GA with no risk factors, urine protein/urine creatinine ratios and/or urine microalbumin/urine creatinine ratios may be obtained at the discretion of the primary provider*.	5
*Imaging*	*We suggest that those at the highest risk (preterm infants* < *28 weeks GA or those born 28 to* < *34 weeks GA but with* ≥ *1 modifying risk factor) may benefit from kidney ultrasound at a two year assessment at the discretion of the ordering nephrologist*.	5
***When should they be followed?***		
*Discharge*	*We recommend all infants* < *34 weeks have an accurate BP obtained, serum creatinine level checked, and parent(s)/guardian(s) provided with kidney health education*.	3
*Kidney Health Assessment at Two Years*	*We recommend all infants* < *34 weeks have follow-up at two years of age, have an accurate BP obtained, serum creatinine level assessed, and parents provided with kidney health education*	3
***How should it be performed?***		
*Kidney Health Education and Resources*	*We recommend that provider education should focus on additional risk factors for CKD beyond preterm birth, strategies to optimize kidney health at NICU discharge and throughout childhood, and monitoring strategies, with an emphasis on the importance of kidney-focused follow-up*	5
*Comprehensive Kidney Assessment*	*BP assessment* *Serum Creatinine and Cystatin C for eGFR calculation* *Urine protein and/or albumin to urine creatinine ratio* * + /- Kidney Ultrasound*	*See above testing*

*AKI*, acute kidney injury, *BP*, blood pressure; *CAKUT*, congenital anomaly of kidney or urinary tract; *CKD*, chronic kidney disease, *eGFR*, estimated glomerular filtration rate, *GA*, gestational age, *HTN*, hypertension, *IUGR*, intrauterine growth restriction, *NICU*, neonatal intensive care unit, *SGA*, small for gestational age.

#### At- risk: preterm neonates 28 to < 34 weeks GA

The risk of CKD in preterm infants born 28–34 weeks GA should not be minimized. A Swedish registry found that children born at 28–33 weeks GA had a sixfold increased CKD risk by 0-9 years, with an adjusted hazard ratio of 5.96 (95% CI 4.07–8.73 *p* < 0.001) compared to term counterparts [6]. Similarly, a Norwegian cohort showed that preterm birth ( < 37 weeks GA) was associated with a 48% increase in the odds of stage 3–5 CKD (OR [95% CI]; 1.48 [1.33–1.66]) by a mean age of 26 years [34]. A recent meta-analysis indicated that individuals aged 6–41 years old who were born preterm had lower eGFR, higher BP, higher urine albumin/urine creatinine ratios, and smaller kidneys [21]. HTN is also a concern, with 13% of infants born <34 weeks GA having elevated systolic blood pressure ( > 90th percentile) at 3-year follow-up and additional studies demonstrating higher BP in later childhood and adulthood after preterm birth [35–37].We recommend infants born 28–34 weeks GA have kidney health education (see section: **What and how should it be performed?**) and a BP assessment at every medical encounter in line with current AAP guidelines (**Level of Evidence 5)**.

#### Modifying risk factors: AKI

Acute kidney injury occurs in up to 30% of hospitalized neonates and is independently associated with increased length of hospital stay and mortality [38, 39]. The risk of AKI is inversely correlated with gestational age and birth weight in preterm neonates and is not well detected or documented [7, 40]. In pediatric and adult patients, AKI is an independent risk factor for subsequent CKD [41–45]. For neonates, the link between AKI and CKD remains less clear. A secondary analysis of the Preterm Epo Neuroprotection Trial (PENUT) found AKI Stage ≥2 was associated with an increased systolic BP at two years of age with an odds ratio of 1.24 (95% CI 1.04-1.48) [20]. In contrast, other investigators have found no association between neonatal AKI with kidney function later in childhood [46]. This lack of association between AKI and the development of HTN and/or CKD was also supported in a retrospective study examining 222 extremely low birth weight (ELBW, <1000 g) infants, 23 of whom had severe AKI [47].

Differences in outcomes, study design, heterogeneity of AKI causes, inconsistent documentation and study duration likely explains the inconsistent findings [40, 48–51]. Until these conflicting results are addressed in large prospective studies, we believe the health community should still consider CKD monitoring in children with a history of AKI in the NICU. We recommend that AKI is defined using modified neonatal KDIGO criteria, with attention paid to accurate diagnosis, documentation, and communication at the time of discharge [17, 50, 52]. Preterm infants (28–34 weeks GA) with AKI should undergo comprehensive kidney health assessment at two years of age in addition to BP assessment and kidney education (**Level of Evidence 3)**.

#### Modifying risk factors: IUGR, SGA, and VLBW

Perinatal growth restriction, including intrauterine or fetal growth restriction (IUGR/FGR) and/or small for gestational age (SGA) ( < 10th percentile weight for gestational age), and very low birthweight (VLBW, <1500 g), are independent risk factors for developing CKD and HTN among infants born at 28–34 weeks GA [34, 36, 53, 54]. In a cohort of 446 VLBW infants followed to 3 years of age, a history of IUGR was associated with a significant reduction in kidney function [55]. The Norwegian Renal Registry described that preterm infants born with a combination of low birth weight ( < 2500 g) and SGA ( < 10th percentile) had an increased adjusted hazard ratio 2.96 (95% CI 1.84–4.76) for kidney failure by age 50 [34]. A separate study following preterm ( ≤ 32 weeks GA) VLBW infants until 10–13 years of age noted that 48% (30/62) had an SBP ≥90th percentile [36]. Additionally, in a cohort of Indian children born at <35 weeks’ gestation and evaluated at 12-18 months, for every one week increase in GA, eGFR increased by 5.48 mL/min/1.73m^2^ [53]. More notably, for every increase in birthweight Z-score by one, eGFR rose by 14.34 mL/min/1.73m^2^ (2.66–26.02) [53]. The direct relationship among GA, birth weight, and decreased eGFR is additionally supported in a Japanese cohort of infants born before 35 weeks GA, who were admitted to the NICU without a history of CAKUT, congenital heart disease, or AKI, and then followed up at 2 years of age [54]. We recommend that preterm infants (28–34 weeks GA) born < 1500 g (VLBW, and SGA at 33 weeks’ GA and <3rd percentile at 34 weeks GA) are at high risk for CKD and should undergo comprehensive kidney health assessment (BP assessment, serum creatinine and cystatin C for eGFR calculation, urine protein and/or albumin to urine creatinine ratio, ±kidney ultrasound) at two years of age in addition to kidney education (**Level of Evidence 3)**.

#### Other modifying risk factors

Attention must be made to maternal history, particularly exposure preeclampsia and tobacco exposure, as these conditions have been associated with kidney disease in offspring [56, 57]. Growing literature supports the role of chronic lung disease (bronchopulmonary dysplasia) in the development of both short and long-term kidney dysfunction [58–61]. However, the relationship is highly confounded, and high-strength literature does not exist to inform recommendations [58]. Preterm infants may have additional risk factors following NICU discharge (Fig. 1B). Hospital readmission is common with nearly 50% of extremely preterm infants being hospitalized during the first two years of life [62–64]. Significant nephrotoxin medication exposures, new or recurrent incidences of AKI, or development of diabetes following NICU discharge can further elevate a child’s risk. In addition, family history and the emerging understanding of inheritable genetic traits are important risk factors in the progression to CKD [65]. Family history, rehospitalization, and other risk factors should be considered when determining an individual’s risk for kidney disease and need for follow-up (**Level of Evidence 4).**

#### Extremely preterm neonates (<28 weeks)

Infants born at earliest gestational ages at the highest risk for CKD [6]. In a Swedish birth registry, the adjusted hazard ratio (HR) for CKD by adulthood in infants born <28 weeks GA was 3.01 (95% CI 1.67–5.45) or an adjusted incidence rate of 13.45 per 100,000 person-years [6]. Other studies have shown that clinical features of CKD were detectable in extremely preterm infants ( < 28 weeks) at two years of age [20, 66]. We recommend that infants born < 28 weeks GA have a two-year (either chronologic or corrected age) comprehensive kidney health assessment. Assessment may occur sooner if additional CKD risk factors are present. (**Level of Evidence 3).**

### When and where should they be followed?

#### Discharge

Screening at hospital discharge is an opportunity to identify those with CKD prior to discharge. Although supporting data is limited, several publications demonstrate that elevated SCr or BP can be detected at discharge [67–69]. Blood pressure should be measured in neonates per unit protocol and compared to published normative values based on post-menstrual age [70]. Preterm infants <34 weeks GA with a SCr>0.5 mg/dl or a BP>95th percentile at rest, nephrocalcinosis, or CAKUT at the time of discharge warrant pediatric nephrology follow-up. A SCr cut off of 0.5 mg/dL was based on consensus and data from Bateman et al. showing that for neonates born 25-27 weeks’ GA, the 95^th^ percentile cut-off for SCr at 60 days of age is 0.51 mg/dL [71]. We recommend all infants < 34 weeks GA have an accurate BP obtained, serum creatinine level assessed, and caregivers provided with kidney health education before NICU discharge (**Level of Evidence 3).**

#### Kidney health monitoring between discharge and two years

In newborns and infants, renal blood flow is physiologically low, gradually normalizing to adult levels by two years of age, when GFR stabilizes [72]. Therefore the best evidence for follow-up to determine subsequent CKD risk is at two years of age. Providing kidney health education at annual primary care visits, along with routine blood pressure screening may ensure follow-up and potentially modify future risk factors such as nephrotoxic medication exposure and aligns with the AAP recommendations related to BP screening [12, 73]. We also recognize that assessment by a pediatric nephrologist may be warranted if additional CKD risk factors or other significant exposures that increase this risk of CKD occur before this visit.

We additionally recognize that a pediatrician or family practice physician may not be every infant’s medical home. In a study published in 2014, two-fifths of complex medical patients did not visit their primary care physician in the previous year [74]. Thus, it is important for all care providers to ensure BPs are obtained and kidney health education is performed at follow-up visits. *We recommend that infants* < *34 weeks GA have kidney health education and BP assessment at every healthcare encounter following discharge*** (Level of Evidence 4)**. We recommend at-risk infants < 34 weeks GA have blood pressures checked at every medical encounter per the AAP recommendations and a follow-up at two years of age with kidney health education and optional kidney health assessment (**Level of Evidence 3)**. We recommend for high-risk infants (<28 weeks or 28 to < 34 weeks with at least one risk factor) they have education provided, an accurate BP obtained and a kidney health assessment. (**Level of Evidence 3)**.

#### Kidney health monitoring after two years

Our recommendations are focused on early childhood based on the current evidence. However, this cohort of patients should be followed throughout childhood as their risk of CKD is likely to increase with age and potential additive risk factors such as obesity, diabetes, chronic illnesses, and nephrotoxic medication exposure. Interestingly, preterm neonates have a greater risk of developing both type 1 and 2 diabetes at a population level and in smaller studies, children born preterm have been found to have insulin insensitivity [75–78]. Diabetes is the leading cause of end-stage kidney disease in the adult population; therefore, those born preterm with an increased risk for diabetes should be monitored closely. It is important to consider kidney monitoring during periods of rapid growth, such as puberty, as kidney function in patients with CKD can deteriorate during this time of accelerated growth and hormonal exposure [79]. Persistent abnormalities in the kidney health assessment should be monitored in accordance with standard monitoring practices established by pediatric nephrologists for albuminuria, elevated blood pressure, hypertension, reduced GFR, or renal hypoplasia.

### What and how should it be performed?

#### Kidney health education and resources

An emphasis on kidney health at NICU discharge and subsequent follow-up with providers is critical–both for families and providers. In a single center study, 75% of caregivers were unaware their infant had AKI and 94% were unaware of any problem with their kidneys [80]. Family education should include education on nephrotoxic medications (including medications like ibuprofen which are over-the-counter) and a discussion of the importance of kidney health awareness (Fig. 1C). As resources vary by institution, this education may be provided by the discharging provider or a subspecialist. Parents identified the transition from the NICU to home as an opportunity to learn to prepare for the unexpected and identified a need for reliable sources of information [81, 82]. Thus, identification of any potential barriers and discussion, preparation, and solutions to overcome them may increase adherence rate for future kidney follow-up [82]. We recognize challenges in providing kidney health education both at discharge and in the outpatient settings due to a variety of constraints. Reliable and easily accessible resources should be provided to families as reference at times when they can accept and retain information. We recommend that education focus on avoiding additional risk factors for CKD beyond preterm birth with an emphasis on the importance of kidney-focused follow-up at 2 years of age **(Level of Evidence 5)**.

#### Comprehensive kidney assessment

For all preterm neonates <34 weeks GA, BP assessment should be performed by the primary care provider as recommended by the AAP with expansion to all patient interactions [73]. To determine if a blood pressure is elevated, for neonates we recommend using the published reference data and for children over the age of 1 year, we recommend the 2017 guidelines published in Pediatrics which also includes details on standardized techniques [12, 83]. *High-risk* neonates ( < 28 weeks GA or those <34 weeks GA with additional risk factors) should undergo a comprehensive kidney evaluation including BP assessment, serum and urine studies as described below. Kidney imaging, such as ultrasound, may help guide kidney assessment, but little evidence exists to support its use. If additional risk factors are present or there are clinical concerns like hypertension or failure to thrive, a comprehensive kidney evaluation can be performed earlier than two years. Kidney health education should accompany all laboratory testing as the risk of progression to CKD in preterm infants extends past two years of age [6].

#### Laboratory studies

Laboratory studies include both blood testing and urine testing. We recommend the measurement of serum creatinine (SCr) and cystatin C to estimate GFR using the U25 formula [12, 73, 84, 85]. The training set for U25 formula included 25 individuals born preterm which was 4% of the total population. Although gold standard, estimated GFR using SCr and cystatin C were not designed for those <1 year of age and should be interpreted with caution for multiple reasons in these populations depending on the time point assessed [66, 86, 87]. An eGFR<90 ml/min/1.73m^2^ would be considered abnormal, CKD stage 2. We recommend that serum cystatin C and serum creatinine should be obtained at two years of age for those < 28 weeks’ GA or in those 29 to < 34 weeks’ GA with modifying risk factors or concerns (**Level of Evidence 3)**.

Proteinuria and albuminuria can be early signs of kidney damage and may be a harbinger for the development of CKD [88–92]. Quantitation of urine protein detects all proteins in the urine, while urine albumin is more specific for glomerular injury, and the addition of a urine creatinine allows assessment of the concentration of the sample. Accepted cutoffs for normal urine protein to urine creatinine (UPC) in infants are higher (0.81 mg of protein per mg of creatinine) until 6 months, 0.5 mg/mg from 6-24 months and <0.2 mg/mg after 24 months. Normal urine albumin to urine creatinine ratios (UAC) is <30 mg albumin per gram of creatinine. *Micro*albuminuria, referring to smaller amounts of albuminuria (30–300 mg of albumin to g of creatinine) has been linked to kidney disease progression and thus was included in the cutoffs above [93, 94]. These ratios can be challenging and inaccurate in the setting of low urine creatinine, falsely increasing a UPC value. Given the increased risk of kidney disease of this population, we suggest that proteinuria and/or albuminuria at two years of age is an opportunity for screening and/or early detection of CKD. We recommend that those at high-risk (preterm infants < 28 weeks GA or those born 28 to < 34 weeks GA but with ≥ 1 modifying risk factor) have urine albumin to creatinine ratios at a two-year follow-up visit. For infants 28 weeks GA to < 34 weeks GA with no risk factors, urine protein/creatinine and/or urine albumin/creatinine ratio may be obtained at the discretion of the primary provider. (**Level of Evidence 5)**.

#### Imaging

The impact of kidney volume as a marker for the risk of subsequent chronic kidney disease in preterm neonates remains a hot topic of research. Studies evaluating the association of kidney size with progression to CKD is limited primarily to children with anomalies of the kidney and urinary tract [95, 96]. In early adulthood (median age 23 years), those born <30 weeks GA had lower kidney volumes than matched controls born at term [97]. Smaller kidney volumes and lengths have also been observed in cohorts of adolescents born with IUGR and <30 weeks GA [98], and 12-18 month-old children, born preterm and SGA [53]. Normative values for kidney length and volume have been published to guide interpretation in infants and children, providing important context when assessing for abnormal growth [99, 100]. Thus, evaluation of kidney size over time may help predict CKD. Further research is greatly needed to better understand kidney volume as a risk factor for subsequent CKD [101, 102]. We suggest that those at the highest risk (preterm infants < 28 weeks GA or those born 28 to < 34 weeks GA but with ≥ 1 modifying risk factor) may benefit from kidney ultrasound at a two-year assessment at the discretion of the ordering nephrologist (**Level of Evidence 5)**.

#### Gaps in knowledge

Despite the evidence above, many recommendations are limited given the significant gaps in the literature. Important gaps in knowledge remain and we recognize that the recommendations above serve only as a starting point. The landscape on which this framework was built is rapidly evolving as younger gestational-age infants survive into early adulthood. Advocating for the inclusion of both short and long-term kidney outcomes in all NICU research studies will help to start to fill this gap [103].

We note that it is essential to standardize outcome measures and gather meaningful data to related to screening, efficiency, and impact through multi-center longitudinal studies [103]. Furthermore, while AKI is well understood to be a risk factor for CKD, AKI encompasses a heterogeneous group of mechanisms and represents functional loss defined by imperfect biomarkers [17]. Thus, improvement in phenotyping of AKI is necessary to aid in risk stratification of progression to CKD for potential targeted prevention [17]. Risk stratification includes diagnostic tools and clinical data. Serum creatinine, cystatin C, and urine output are the most well-studied, but remain largely imperfect for both neonates and infants [48]. Urinary biomarkers, such as urine neutrophil gelatinase-associated lipocalin is gaining traction and implementation, but has not yet been used for long-term risk stratification [104–106]. Further study is greatly needed utilizing current diagnostic tools, clinical data, and novel biomarkers to optimize risk stratification to guide future follow-up strategies.

#### Research priorities

Research needed includes mechanistic, clinical, and implementation studies to prioritize the outlined knowledge gaps. Statistical models and risk score calculators will be critical for rapid identification, streamlining referral and follow-up scheduling, and enhancing overall implementation and adherence to recommendations. Neonatal kidney health registries and long-term follow-up are urgently needed to understand both in-hospital risk factors, risk modifiers following discharge, and ultimate progression to CKD. Additionally, as kidney replacement devices for infants become more readily available and potentially more widely used, the role of dialysis on long-term kidney health will be critical to address.

## Discussion (feasibility, implementation challenges, and potential benefits and harms)

These consensus recommendations provide cohesive best practices in a population at high risk for kidney disease based on current evidence and were developed in a consensus manner with multi-disciplinary experts in the field. These recommendations provide a framework to begin to study and optimize the follow-up of these high-risk neonates.

We recognize that our recommendations present implementation challenges. We estimated that ~20,000 preterm infants would be considered at high risk of future CKD [107]. While a two-year follow-up visit may be most beneficial with a pediatric nephrologist, current workforce issues and uneven distribution of pediatric nephrologists in the country may make this unfeasible [108]. With the expansion of physician extenders like nurse practitioners and physician assistants, and the establishment of specific neonatal AKI follow-up clinics, it may be possible for more children to see a Pediatric Nephrology team in future recommendations. Also, since our recommendations require knowing the potential risk factors that change follow-up timing and screening, it will be vitally important that risk factors like SGA and AKI are included in discharge summaries. Future work could be to find ways to add these risk factors to other enduring childhood health documentation like vaccination or growth records.

We note that the BP recommendations align with the AAP guidelines for screening after NICU discharge. We acknowledge that outpatient pediatricians may not have the necessary equipment for accurate assessment of infant BPs. However, advocacy is needed to ensure that appropriate blood pressure cuffs are available in all pediatric practices. Given the large number of infants who will require blood pressure monitoring including, but not limited to, NICU graduates, ensuring access to proper tools must be a priority.

The recommendations confer several benefits but also risks to consider. The main risk is an increase in anxiety for the parents of these already medically complex children. The potential benefits of these recommendations are significant. Early screening may detect signs of CKD months to years earlier than it would have been identified without screening. This early detection could result in earlier control of blood pressure, treatment of proteinuria, and could ultimately slow the progression of CKD. The educational component could result in parents feeling more empowered and informed and result in avoidance of nephrotoxic medications and maintenance of a healthy weight. From a neonatology standpoint, these recommendations could continue to inform the large work force of the importance of the kidney in NICU care and continue to raise awareness about the implications of preterm birth and AKI on long-term kidney health. Our group recognizes the potential harm that may result from these recommendations, including the potential for more blood draws, BP measurements, and follow up including an increased burden to an already stretched work force in pediatric nephrology [107].

Preterm infants, especially those born before 34 weeks GA, have an increased risk of CKD and HTN. As neonatal survival improves, the long-term adverse impact of preterm birth on kidney health may increase. Through the development these consensus recommendations for kidney health monitoring, we provide guidance on kidney health assessments at NICU discharge and at two years of age for at-risk infants. Comprehensive screening and kidney education for families are crucial to early detection and the management of kidney disease. More research is needed to fill the gaps in knowledge, modifiable risk factors, risk stratification and the interplay between kidney health and other organ systems. These recommendations represent an important step toward improving outcomes for this high-risk population.

## Supplementary information

Supplemental Material 1

## References

[1] Ehret DEY, Edwards EM, Greenberg LT, Bernstein IM, Buzas JS, Soll RF, et al. Association of antenatal steroid exposure with survival among infants receiving postnatal life support at 22 to 25 weeks’ gestation. JAMA Netw Open. 2018;1:e183235. 30646235 10.1001/jamanetworkopen.2018.3235 PMC6324435

[2] Msall ME. The limits of viability and the uncertainty of neuroprotection: challenges in optimizing outcomes in extreme prematurity. Pediatrics. 2007;119:158–60. 17200283 10.1542/peds.2006-3095

[3] Elgin TG, Berger JN, Kaluarachchi DC, Dagle JM, Thomas B, Colaizy TT, et al. Outcomes of infants with birthweights less than 501 g compared to infants weighing 501-750 g at a center utilizing first intention high frequency jet ventilation. Front Pediatr. 2024;12:1392079. 39315359 10.3389/fped.2024.1392079 PMC11416967

[4] White SL, Perkovic V, Cass A, Chang CL, Poulter NR, Spector T, et al. Is low birth weight an antecedent of CKD in later life? A systematic review of observational studies. Am J Kidney Dis. 2009;54:248–61. 19339091 10.1053/j.ajkd.2008.12.042

[5] Greenbaum LA, Munoz A, Schneider MF, Kaskel FJ, Askenazi DJ, Jenkins R, et al. The association between abnormal birth history and growth in children with CKD. Clin J Am Soc Nephrol. 2011;6:14–21. 21030583 10.2215/CJN.08481109 PMC3022235

[6] Crump C, Sundquist J, Winkleby MA, Sundquist K. Preterm birth and risk of chronic kidney disease from childhood into mid-adulthood: national cohort study. BMJ. 2019;365:l1346. 31043374 10.1136/bmj.l1346 PMC6490674

[7] Carmody JB, Charlton JR. Short-term gestation, long-term risk: prematurity and chronic kidney disease. Pediatrics. 2013;131:1168–79. 23669525 10.1542/peds.2013-0009

[8] Jetton JG, Boohaker LJ, Sethi SK, Wazir S, Rohatgi S, Soranno DE, et al. Incidence and outcomes of neonatal acute kidney injury (AWAKEN): a multicentre, multinational, observational cohort study. Lancet Child Adolesc Health. 2017;1:184–94. 29732396 10.1016/S2352-4642(17)30069-X PMC5933049

[9] Rumpel JA, Perazzo S, Bona J, South AM, Harer MW, Liu D, et al. ADVANCE: a biomedical informatics approach to investigate acute kidney injury in infants. Pediatr Res. 2025;97:608–13. 10.1038/s41390-024-03436-5 PMC12024515 39122822

[10] Flynn RS, Zedalis J, Denburg MR, Bernbaum JC, DeMauro SB. Improving blood pressure screening in neonatal follow-up clinic: a quality improvement initiative. Pediatr Qual Saf. 2022;7:e559. 35720869 10.1097/pq9.0000000000000559 PMC9197357

[11] Shah L, Hossain J, Xie S, Zaritsky J. Poor adherence to early childhood blood pressure measurement guidelines in a large pediatric healthcare system. Pediatr Nephrol. 2019;34:697–701. 30406366 10.1007/s00467-018-4132-y

[12] Flynn JT, Kaelber DC, Baker-Smith CM, Blowey D, Carroll AE, Daniels SR, et al. Clinical practice guideline for screening and management of high blood pressure in children and adolescents. Pediatrics. 2018;142:e20181739. 10.1542/peds.2017-1904 28827377

[13] Kidney Disease: Improving Global Outcomes CKDWG. KDIGO 2024 clinical practice guideline for the evaluation and management of chronic kidney disease. Kidney Int. 2024;105:S117–314. 38490803 10.1016/j.kint.2023.10.018

[14] Burke BJ, Liu W, Worley S, Bou Matar RN. Abstract P194: Compliance With 2017 AAP guidelines for childhood hypertension: diagnosis and management of pediatric hypertension. Hypertension. 2020;76:AP194–AP.

[15] Starr MC, Harer MW, Steflik HJ, Gorga S, Ambalavanan N, Beck TM, et al. Kidney health monitoring in neonatal intensive care unit graduates: a modified delphi consensus statement. JAMA Netw Open. 2024;7:e2435043. 39269711 10.1001/jamanetworkopen.2024.35043 PMC12163980

[16] Bellomo R, Ronco C, Kellum JA, Mehta RL, Palevsky P. Acute dialysis quality initiative w. acute renal failure - definition, outcome measures, animal models, fluid therapy and information technology needs: the Second International Consensus Conference of the Acute Dialysis Quality Initiative (ADQI) Group. Crit Care. 2004;8:R204–12. 15312219 10.1186/cc2872 PMC522841

[17] Goldstein SL, Akcan-Arikan A, Alobaidi R, Askenazi DJ, Bagshaw SM, Barhight M, et al. Consensus-based recommendations on priority activities to address acute kidney injury in children: a modified Delphi consensus statement. JAMA Netw Open. 2022;5:e2229442. 36178697 10.1001/jamanetworkopen.2022.29442 PMC9756303

[18] Ogrinc G, Davies L, Goodman D, Batalden P, Davidoff F, Stevens D. Squire 2.0 (Standards for Quality Improvement Reporting Excellence): revised publication guidelines from a detailed consensus process. Am J Crit Care. 2015;24:466–73. 26523003 10.4037/ajcc2015455

[19] Hsu CW, Yamamoto KT, Henry RK, De Roos AJ, Flynn JT. Prenatal risk factors for childhood CKD. J Am Soc Nephrol. 2014;25:2105–11. 24744441 10.1681/ASN.2013060582 PMC4147970

[20] Hingorani S, Schmicker R, Ahmad KA, Frantz ID, Mayock DE, La Gamma EF, et al. Prevalence and risk factors for kidney disease and elevated BP in 2-year-old children born extremely premature. Clin J Am Soc Nephrol. 2022;17:1129–38. 35853728 10.2215/CJN.15011121 PMC9435989

[21] Heo JS, Lee JM. The long-term effect of preterm birth on renal function: a meta-analysis. Int J Environ Res Public Health. 2021;18:2951. 10.3390/ijerph18062951 PMC8001027 33805740

[22] Jetton JG, Askenazi DJ. Acute kidney injury in the neonate. Clin Perinatol. 2014;41:487–502. 25155722 10.1016/j.clp.2014.05.001

[23] Hinchliffe SA, Sargent PH, Howard CV, Chan YF, van Velzen D. Human intrauterine renal growth expressed in absolute number of glomeruli assessed by the disector method and Cavalieri principle. Lab Invest. 1991;64:777–84. 2046329

[24] Chevalier RL. Bioenergetic evolution explains prevalence of low nephron number at birth: risk factor for CKD. Kidney360. 2020;1:863–79. 35372951 10.34067/KID.0002012020 PMC8815749

[25] Charlton JR, Springsteen CH, Carmody JB. Nephron number and its determinants in early life: a primer. Pediatr Nephrol. 2014;29:2299–308. 24488483 10.1007/s00467-014-2758-y

[26] Perl AJ, Schuh MP, Kopan R. Regulation of nephron progenitor cell lifespan and nephron endowment. Nat Rev Nephrol. 2022;18:683–95. 36104510 10.1038/s41581-022-00620-w PMC11078284

[27] Callaway DA, McGill-Vargas LL, Quinn A, Jordan JL, Winter LA, Anzueto D, et al. Prematurity disrupts glomeruli development, whereas prematurity and hyperglycemia lead to altered nephron maturation and increased oxidative stress in newborn baboons. Pediatr Res. 2018;83:702–11. 29166383 10.1038/pr.2017.290 PMC5902650

[28] Rodriguez MM, Gomez AH, Abitbol CL, Chandar JJ, Duara S, Zilleruelo GE. Histomorphometric analysis of postnatal glomerulogenesis in extremely preterm infants. Pediatr Dev Pathol. 2004;7:17–25. 15255031 10.1007/s10024-003-3029-2

[29] Sutherland MR, Gubhaju L, Moore L, Kent AL, Dahlstrom JE, Horne RS, et al. Accelerated maturation and abnormal morphology in the preterm neonatal kidney. J Am Soc Nephrol. 2011;22:1365–74. 21636639 10.1681/ASN.2010121266 PMC3137584

[30] Carpenter J, Yarlagadda S, VandenHeuvel KA, Ding L, Schuh MP. Human nephrogenesis can persist beyond 40 postnatal days in preterm infants. Kidney Int Rep. 2024;9:436–50. 38344733 10.1016/j.ekir.2023.10.032 PMC10851065

[31] Gubhaju L, Sutherland MR, Yoder BA, Zulli A, Bertram JF, Black MJ. Is nephrogenesis affected by preterm birth? Studies in a non-human primate model. Am J Physiol Ren Physiol. 2009;297:F1668–77. 10.1152/ajprenal.00163.2009 PMC2801336 19759270

[32] Sutherland MR, Yoder BA, McCurnin D, Seidner S, Gubhaju L, Clyman RI, et al. Effects of ibuprofen treatment on the developing preterm baboon kidney. Am J Physiol Ren Physiol. 2012;302:F1286–92. 10.1152/ajprenal.00216.2011 PMC3362063 22357916

[33] Lee YQ, Beckett EL, Sculley DV, Rae KM, Collins CE, Pringle KG. Relationship between maternal global nutrient restriction during pregnancy and offspring kidney structure and function: a systematic review of animal studies. Am J Physiol Ren Physiol. 2019;316:F1227–35. 10.1152/ajprenal.00082.2019 30969805

[34] Gjerde A, Lillas BS, Marti HP, Reisaeter AV, Vikse BE. Intrauterine growth restriction, preterm birth and risk of end-stage renal disease during the first 50 years of life. Nephrol Dial Transpl. 2020;35:1157–63. 10.1093/ndt/gfaa001 PMC7417009 32040151

[35] Reis JD, Heyne R, Rosenfeld CR, Caraig M, Brown LS, Burchfield PJ, et al. Follow-up of a randomized trial optimizing neonatal nutrition in preterm very low birthweight infants: growth, serum adipokines, renal function and blood pressure. J Perinatol. 2024;44:78–86. 37964083 10.1038/s41372-023-01821-2

[36] Wickland J, Steven Brown L, Blanco V, Heyne R, Turer C, Rosenfeld CR. Persistent high blood pressure and renal dysfunction in preterm infants during childhood. Pediatr Res. 2023;93:217–25. 35484228 10.1038/s41390-022-02083-y

[37] Branda JIF, de Almeida-Pititto B, Bensenor I, Lotufo PA, Ferreira SRG, Brasil E. Associations of prematurity and low birth weight with blood pressure and kidney function in middle-aged participants of the Brazilian Longitudinal Study of Adult Health: ELSA-Brasil. J Nephrol. 2023;36:1373–82. 36646972 10.1007/s40620-022-01549-w

[38] Jetton JG, Guillet R, Askenazi DJ, Dill L, Jacobs J, Kent AL, et al. Assessment of Worldwide Acute Kidney Injury Epidemiology in Neonates: Design of a Retrospective Cohort Study. Front Pediatr. 2016;4:68. 27486571 10.3389/fped.2016.00068 PMC4950470

[39] Meena J, Kumar J, Kocharlakota JP, Gupta H, Mittal P, Kumar A, et al. Acute kidney injury in neonates: a meta-analysis. Pediatrics. 2024;154:e2023065182. 10.1542/peds.2023-065182 38872621

[40] Starr MC, Kula A, Lieberman J, Menon S, Perkins AJ, Lam T, et al. The impact of increased awareness of acute kidney injury in the Neonatal Intensive Care Unit on acute kidney injury incidence and reporting: results of a retrospective cohort study. J Perinatol. 2020;40:1301–7. 32681064 10.1038/s41372-020-0725-y PMC7442645

[41] Sigurjonsdottir VK, Chaturvedi S, Mammen C, Sutherland SM. Pediatric acute kidney injury and the subsequent risk for chronic kidney disease: is there cause for alarm? Pediatr Nephrol. 2018;33:2047–55. 29374316 10.1007/s00467-017-3870-6

[42] Kurzhagen JT, Dellepiane S, Cantaluppi V, Rabb H. AKI: an increasingly recognized risk factor for CKD development and progression. J Nephrol. 2020;33:1171–87. 32651850 10.1007/s40620-020-00793-2

[43] Mammen C, Al Abbas A, Skippen P, Nadel H, Levine D, Collet JP, et al. Long-term risk of CKD in children surviving episodes of acute kidney injury in the intensive care unit: a prospective cohort study. Am J Kidney Dis. 2012;59:523–30. 22206744 10.1053/j.ajkd.2011.10.048

[44] Palant C, Amdur R, Chawla LS. Acute Kidney Injury and CKD: No Respite for a Weary Kidney. Am J Kidney Dis. 2015;66:552–4. 26408233 10.1053/j.ajkd.2015.08.014

[45] Ferenbach DA, Bonventre JV. Mechanisms of maladaptive repair after AKI leading to accelerated kidney ageing and CKD. Nat Rev Nephrol. 2015;11:264–76. 25643664 10.1038/nrneph.2015.3 PMC4412815

[46] Bruel A, Roze JC, Quere MP, Flamant C, Boivin M, Roussey-Kesler G, et al. Renal outcome in children born preterm with neonatal acute renal failure: IRENEO-a prospective controlled study. Pediatr Nephrol. 2016;31:2365–73. 27335060 10.1007/s00467-016-3444-z

[47] Maqsood S, Fung N, Chowdhary V, Raina R, Mhanna MJ. Outcome of extremely low birth weight infants with a history of neonatal acute kidney injury. Pediatr Nephrol. 2017;32:1035–43. 28194575 10.1007/s00467-017-3582-y

[48] Askenazi D. Are we ready for the clinical use of novel acute kidney injury biomarkers? Pediatr Nephrol. 2012;27:1423–5. 22689085 10.1007/s00467-012-2185-x

[49] Charlton JR, Boohaker L, Askenazi D, Brophy PD, D’Angio C, Fuloria M, et al. Incidence and risk factors of early onset neonatal AKI. Clin J Am Soc Nephrol. 2019;14:184–95. 31738181 10.2215/CJN.03670318 PMC6390916

[50] Chmielewski J, Chaudhry PM, Harer MW, Menon S, South AM, Chappell A, et al. Documentation of acute kidney injury at discharge from the neonatal intensive care unit and role of nephrology consultation. J Perinatol. 2022;42:930–6. 35676535 10.1038/s41372-022-01424-3 PMC9280854

[51] Starr MC, Chaudhry P, Brock A, Vincent K, Twombley K, Bonachea EM, et al. Improving the identification of acute kidney injury in the neonatal ICU: three centers’ experiences. J Perinatol. 2022;42:243–6. 34480111 10.1038/s41372-021-01198-0

[52] Jetton JG, Askenazi DJ. Update on acute kidney injury in the neonate. Curr Opin Pediatr. 2012;24:191–6. 22227783 10.1097/MOP.0b013e32834f62d5 PMC5545784

[53] Reddy KV, Pawale D, Shah M, Mouli D, Murki S. Assessment of renal growth and function in preterm infants at corrected age of 12-18 month. Indian Pediatr. 2020;57:411–4. 32444513

[54] Horie A, Abe Y, Koike D, Hirade T, Nariai A, Ito T, et al. Long-term renal follow up of preterm neonates born before 35 weeks of gestation. Pediatr Int. 2019;61:1244–9. 31495051 10.1111/ped.14004 PMC6973113

[55] Uemura O, Ishikura K, Kaneko T, Hirano D, Hamasaki Y, Ogura M, et al. Perinatal factors contributing to chronic kidney disease in a cohort of Japanese children with very low birth weight. Pediatr Nephrol. 2021;36:953–60. 33068163 10.1007/s00467-020-04791-1 PMC7910374

[56] Lihme I, Basit S, Lihme F, Damholt MB, Hjorth S, Nohr EA, et al. A nationwide register-based cohort study examined the association between preeclampsia in mothers and the risk of kidney disease in their offspring. Kidney Int. 2025;108:283–92. 40383228 10.1016/j.kint.2025.04.017

[57] Taal HR, Geelhoed JJ, Steegers EA, Hofman A, Moll HA, Lequin M, et al. Maternal smoking during pregnancy and kidney volume in the offspring: the Generation R Study. Pediatr Nephrol. 2011;26:1275–83. 21617916 10.1007/s00467-011-1848-3 PMC3119805

[58] Wallace SW, Geers ER, Niehaus JZ, Cristea AI, Starr MC. Kidney complications in children with bronchopulmonary dysplasia. Pediatr Res. 2025;97:2431–35. 10.1038/s41390-024-03638-x PMC12463462 39443697

[59] Starr MC, Griffin R, Gist KM, Segar JL, Raina R, Guillet R, et al. Association of fluid balance with short- and long-term respiratory outcomes in extremely premature neonates: a secondary analysis of a randomized clinical trial. JAMA Netw Open. 2022;5:e2248826. 36580332 10.1001/jamanetworkopen.2022.48826 PMC9856967

[60] Starr MC, Boohaker L, Eldredge LC, Menon S, Griffin R, Mayock DE, et al. Acute kidney injury and bronchopulmonary dysplasia in premature neonates born less than 32 weeks’ gestation. Am J Perinatol. 2020;37:341–8. 31777046 10.1055/s-0039-3400311 PMC7409513

[61] Starr MC, Wilson AC. Systemic hypertension in infants with bronchopulmonary dysplasia. Curr Hypertens Rep. 2022;24:193–203. 35266097 10.1007/s11906-022-01179-4

[62] Ambalavanan N, Carlo WA, McDonald SA, Yao Q, Das A, Higgins RD, et al. Identification of extremely premature infants at high risk of rehospitalization. Pediatrics. 2011;128:e1216–25. 22007016 10.1542/peds.2011-1142 PMC3208965

[63] Ray KN, Lorch SA. Hospitalization of early preterm, late preterm, and term infants during the first year of life by gestational age. Hosp Pediatr. 2013;3:194–203. 24313087 10.1542/hpeds.2012-0063

[64] Smith VC, Zupancic JA, McCormick MC, Croen LA, Greene J, Escobar GJ, et al. Rehospitalization in the first year of life among infants with bronchopulmonary dysplasia. J Pediatr. 2004;144:799–803. 15192629 10.1016/j.jpeds.2004.03.026

[65] Harshman LA, Zepeda-Orozco D. Genetic considerations in pediatric chronic kidney disease. J Pediatr Genet. 2016;5:43–50. 27617141 10.1055/s-0035-1557111 PMC4918706

[66] Harer MW, Pope CF, Conaway MR, Charlton JR. Follow-up of acute kidney injury in neonates during childhood years (FANCY): a prospective cohort study. Pediatr Nephrol. 2017;32:1067–76. 28255805 10.1007/s00467-017-3603-x

[67] Shalaby MA, Sawan ZA, Nawawi E, Alsaedi S, Al-Wassia H, Kari JA. Incidence, risk factors, and outcome of neonatal acute kidney injury: a prospective cohort study. Pediatr Nephrol. 2018;33:1617–24. 29869723 10.1007/s00467-018-3966-7

[68] Gallo D, de Bijl-Marcus KA, Alderliesten T, Lilien M, Groenendaal F. Early acute kidney injury in preterm and term neonates: incidence, outcome, and associated clinical features. Neonatology. 2021;118:174–9. 33780939 10.1159/000513666

[69] Kraut EJ, Boohaker LJ, Askenazi DJ, Fletcher J, Kent AL, Neonatal Kidney C. Incidence of neonatal hypertension from a large multicenter study [Assessment of Worldwide Acute Kidney Injury Epidemiology in Neonates-AWAKEN]. Pediatr Res. 2018;84:279–89. 29795211 10.1038/s41390-018-0018-8

[70] Harer MW, Kent AL. Neonatal hypertension: an educational review. Pediatr Nephrol. 2019;34:1009–18. 29974208 10.1007/s00467-018-3996-1

[71] Bateman DA, Thomas W, Parravicini E, Polesana E, Locatelli C, Lorenz JM. Serum creatinine concentration in very-low-birth-weight infants from birth to 34-36 wk postmenstrual age. Pediatr Res. 2015;77:696–702. 25675426 10.1038/pr.2015.25 PMC4407015

[72] Heilbron DC, Holliday MA, al-Dahwi A, Kogan BA. Expressing glomerular filtration rate in children. Pediatr Nephrol. 1991;5:5–11. 2025537 10.1007/BF00852829

[73] Hauk L. Screening and management of High BP in children and adolescents: an updated guideline from the AAP. Am Fam Physician. 2018;97:543–4. 29671487

[74] Berry JG, Hall M, Neff J, Goodman D, Cohen E, Agrawal R, et al. Children with medical complexity and Medicaid: spending and cost savings. Health Aff (Millwood). 2014;33:2199–206. 25489039 10.1377/hlthaff.2014.0828 PMC5164920

[75] Crump C. An overview of adult health outcomes after preterm birth. Early Hum Dev. 2020;150:105187. 32948365 10.1016/j.earlhumdev.2020.105187 PMC7480736

[76] Li S, Zhang M, Tian H, Liu Z, Yin X, Xi B. Preterm birth and risk of type 1 and type 2 diabetes: systematic review and meta-analysis. Obes Rev. 2014;15:804–11. 25073871 10.1111/obr.12214

[77] Regan FM, Cutfield WS, Jefferies C, Robinson E, Hofman PL. The impact of early nutrition in premature infants on later childhood insulin sensitivity and growth. Pediatrics. 2006;118:1943–9. 17079565 10.1542/peds.2006-0733

[78] Hofman PL, Regan F, Jackson WE, Jefferies C, Knight DB, Robinson EM, et al. Premature birth and later insulin resistance. N Engl J Med. 2004;351:2179–86. 15548778 10.1056/NEJMoa042275

[79] Ardissino G, Testa S, Dacco V, Paglialonga F, Vigano S, Felice-Civitillo C, et al. Puberty is associated with increased deterioration of renal function in patients with CKD: data from the ItalKid Project. Arch Dis Child. 2012;97:885–8. 22833407 10.1136/archdischild-2011-300685

[80] Starr MC, Vanderkolk J, Goswami S, Slagle CL, Soranno DE. Caregiver awareness and knowledge of acute kidney injury in hospitalized children. JAMA Netw Open. 2024;7:e2442442. 39480426 10.1001/jamanetworkopen.2024.42442 PMC11528335

[81] Berman L, Raval MV, Ottosen M, Mackow AK, Cho M, Goldin AB. Parent perspectives on readiness for discharge home after neonatal intensive care unit admission. J Pediatr. 2019;205:98–104.e4. 30291021 10.1016/j.jpeds.2018.08.086

[82] Starr MC, Wallace S, Moore C, Cockrum B, Hawryluk B, Carroll A, et al. Development of a family-centered communication tool for kidney health in premature infants: qualitative focus group study using human-centered design methodology. J Particip Med. 2023;15:e45316. 37428553 10.2196/45316 PMC10366965

[83] Kent AL, Chaudhari T. Determinants of neonatal blood pressure. Curr Hypertens Rep. 2013;15:426–32. 23917808 10.1007/s11906-013-0375-y

[84] Dionne JM, Flynn JT. Management of severe hypertension in the newborn. Arch Dis Child. 2017;102:1176–9. 28739634 10.1136/archdischild-2015-309740

[85] Pierce CB, Munoz A, Ng DK, Warady BA, Furth SL, Schwartz GJ. Age- and sex-dependent clinical equations to estimate glomerular filtration rates in children and young adults with chronic kidney disease. Kidney Int. 2021;99:948–56. 33301749 10.1016/j.kint.2020.10.047 PMC9083470

[86] Roussel M, Bacchetta J, Sellier-Leclerc AL, Lemoine S, De Mul A, Derain Dubourg L. Creatinine-based formulas are not ideal to estimate glomerular filtration rate in selected pediatric patients: data from a tertiary pediatric nephrology center. Pediatr Nephrol. 2024;39:3023–36. 38884786 10.1007/s00467-023-06275-4

[87] Ibrahim RB, Srivaths P, Tam E, Devaraj S. Utility of cystatin C-based equation for the estimation of glomerular filtration rate in a pediatric population. J Appl Lab Med. 2024;9:803–8. 38656545 10.1093/jalm/jfae034

[88] Konstantelos N, Banh T, Patel V, Vasilevska-Ristovska J, Borges K, Hussain-Shamsy N, et al. Association of low birth weight and prematurity with clinical outcomes of childhood nephrotic syndrome: a prospective cohort study. Pediatr Nephrol. 2019;34:1599–605. 30976899 10.1007/s00467-019-04255-1

[89] Hodgin JB, Rasoulpour M, Markowitz GS, D’Agati VD. Very low birth weight is a risk factor for secondary focal segmental glomerulosclerosis. Clin J Am Soc Nephrol. 2009;4:71–6. 19019999 10.2215/CJN.01700408 PMC2615706

[90] Ikezumi Y, Suzuki T, Karasawa T, Yamada T, Hasegawa H, Nishimura H, et al. Low birthweight and premature birth are risk factors for podocytopenia and focal segmental glomerulosclerosis. Am J Nephrol. 2013;38:149–57. 23920104 10.1159/000353898

[91] Chen CC, Yu T, Chou HH, Chiou YY, Kuo PL. Premature birth carries a higher risk of nephrotic syndrome: a cohort study. Sci Rep. 2021;11:20639. 34667222 10.1038/s41598-021-00164-2 PMC8526683

[92] Fuhrman DY, Schneider MF, Dell KM, Blydt-Hansen TD, Mak R, Saland JM, et al. Albuminuria, Proteinuria, and Renal Disease Progression in Children with CKD. Clin J Am Soc Nephrol. 2017;12:912–20. 28546440 10.2215/CJN.11971116 PMC5460717

[93] Dunger DB, Schwarze CP, Cooper JD, Widmer B, Neil HA, Shield J, et al. Can we identify adolescents at high risk for nephropathy before the development of microalbuminuria? Diabet Med. 2007;24:131–6. 17257274 10.1111/j.1464-5491.2006.02047.x

[94] Steinke JM, Sinaiko AR, Kramer MS, Suissa S, Chavers BM, Mauer M, et al. The early natural history of nephropathy in Type 1 Diabetes: III. Predictors of 5-year urinary albumin excretion rate patterns in initially normoalbuminuric patients. Diabetes. 2005;54:2164–71. 15983218 10.2337/diabetes.54.7.2164

[95] Viteri B, Elsingergy M, Roem J, Ng D, Warady B, Furth S, et al. Ultrasound-based renal parenchymal area and kidney function decline in infants with congenital anomalies of the kidney and urinary tract. Semin Nephrol. 2021;41:427–33. 34916003 10.1016/j.semnephrol.2021.09.004 PMC9036416

[96] Odeh R, Noone D, Bowlin PR, Braga LH, Lorenzo AJ. Predicting risk of chronic kidney disease in infants and young children at diagnosis of posterior urethral valves: initial ultrasound kidney characteristics and validation of parenchymal area as forecasters of renal reserve. J Urol. 2016;196:862–8. 27017936 10.1016/j.juro.2016.03.137

[97] Flahault A, Bollee G, El-Jalbout R, Cloutier A, Santos RAS, Lapeyraque AL, et al. Plasma copeptin is increased and associated with smaller kidney volume in young adults born very preterm. Clin Kidney J. 2022;15:709–17. 35371457 10.1093/ckj/sfab226 PMC8967663

[98] Liefke J, Heijl C, Steding-Ehrenborg K, Morsing E, Arheden H, Ley D, et al. Fetal growth restriction followed by very preterm birth is associated with smaller kidneys but preserved kidney function in adolescence. Pediatr Nephrol. 2023;38:1855–66. 36409369 10.1007/s00467-022-05785-x PMC10154253

[99] Akhavan A, Brajtbord JS, McLeod DJ, Kabarriti AE, Rosenberg HK, Stock JA. Simple, age-based formula for predicting renal length in children. Urology. 2011;78:405–10. 21459422 10.1016/j.urology.2011.01.008

[100] Dinkel E, Orth S, Dittrich M, Schulte-Wissermann H. Renal sonography in the differentiation of upper from lower urinary tract infection. AJR Am J Roentgenol. 1986;146:775–80. 3513490 10.2214/ajr.146.4.775

[101] Licurse A, Kim MC, Dziura J, Forman HP, Formica RN, Makarov DV, et al. Renal ultrasonography in the evaluation of acute kidney injury: developing a risk stratification framework. Arch Intern Med. 2010;170:1900–7. 21098348 10.1001/archinternmed.2010.419

[102] Branagan A, Costigan CS, Stack M, Slagle C, Molloy EJ. Management of Acute Kidney Injury in Extremely Low Birth Weight Infants. Front Pediatr. 2022;10:867715. 35433560 10.3389/fped.2022.867715 PMC9005741

[103] Reidy KJ, Guillet R, Selewski DT, Defreitas M, Stone S, Starr MC, et al. Advocating for the inclusion of kidney health outcomes in neonatal research: best practice recommendations by the Neonatal Kidney Collaborative. J Perinatol. 2024;44:1863–73. 38969825 10.1038/s41372-024-02030-1 PMC11606916

[104] Stoops C, Gavigan H, Krallman K, Anderson N, Griffin R, Slagle C, et al. The utility of urinary NGAL as an alternative for serum creatinine to detect acute kidney injury in infants exposed to nephrotoxic medications in the neonatal intensive care unit. Neonatology. 2024;121:203–12. 10.1159/000535322 PMC10987269 38151013

[105] Slagle CL, Hemmelgarn T, Gavigan HW, Krallman KA, Goldstein SL. Use of urine neutrophil gelatinase-associated lipocalin for nephrotoxic medication acute kidney injury screening in neonates. J Perinatol. 2024;44:1780–5. 38514742 10.1038/s41372-024-01922-6

[106] Marcello M, Virzi GM, Mucino-Bermejo MJ, Milan Manani S, Giavarina D, Salvador L, et al. Subclinical AKI and clinical outcomes in elderly patients undergoing cardiac surgery: diagnostic utility of NGAL versus standard creatinine increase criteria. Cardiorenal Med. 2022;12:94–105. 35661656 10.1159/000525221

[107] Soranno DE, Amaral S, Ashoor I, Atkinson MA, Barletta GM, Braun MC, et al. Responding to the workforce crisis: consensus recommendations from the Second Workforce Summit of the American Society of Pediatric Nephrology. Pediatr Nephrol. 2024;39:3609–19. 38976042 10.1007/s00467-024-06410-9 PMC11511730

[108] Weidemann DK, Orr CJ, Norwood V, Brophy P, Leonard MB, Ashoor I. Child health needs and the pediatric nephrology subspecialty workforce: 2020–2040. Pediatrics. 2024;153:e2023063678P. 10.1542/peds.2023-063678P 38300004

