# Supplementary material for: Kidney care at NICU discharge and follow-up recommendations for preterm infants<34 weeks
Source: J Perinatol. 2026 Feb 26;46(3):498–507. doi: 10.1038/s41372-026-02597-x (PMC13008752; doi:10.1038/s41372-026-02597-x)
Supplement: Supplementary file 1 — Supplemental Material 1 [file 41372_2026_2597_MOESM1_ESM.docx]

**Supplement 1**

**Limits:** Humans, English only, last 25 years

**Results:**

Medline through 11/15/23 n = 2,431

Embase through 11/15/23 n = 2,510

Total after de-duplication n = 3,481

**Strategies**

**Notes:** As discussed, for all searches I excluded records with pregnan* or “gestational hypertension” in the titles. I also required most search terms to be present in the title, author keywords, and/or major Medical Subject Headings (e.g. not solely in the abstract). I included review articles and terms and study types that hopefully will focus on outcomes-related literature. However, because of other uses of these terms, I expect a fair number of false negatives.

**Kidney Ovid MEDLINE – Strategy 1**

| **Concept** | **Keywords** | **MeSH** |
| --- | --- | --- |
| Preterm Neonate | (preterm or pre-mature or pre-matures or preterms or prematurity or pre-maturity or preterm or preterms or pre term? or preemie or preemies or premies or premie or VLBW or VLBWI or VLBW-I or VLBWs or LBW or LBWI or LBWs or ELBW or ELBWI or ELBWs).ti,ab,kf. | exp Infant, Preterm/  exp Infant, Low Birth Weight/ |
| Kidney outcomes (e.g. kidney size, kidney function by glomerular filtration rate, tubular function) | (kidney* or renal* or intrarenal or nephr* or glomerul* or tubulopath* or "tubular function").ti,ab,kf. | exp Kidney/physiopathology  exp Kidney/growth & development  exp Kidney Diseases/  exp Kidney Failure, Chronic/  exp “Renal Insufficiency”/  exp “Renal Insufficiency, Chronic”/  exp “Kidney Neoplasms”/  exp “Kidney Cortex Necrosis”/  exp “Glomerular Filtration Rate”/  exp “Kidney Function Tests”/  exp Nephrolithiasis/  exp Renal Replacement Therapy/  exp Renal Elimination/  exp Renal Circulation  exp Kidney Concentrating Ability/ |
| Follow up/Outcomes | (outcome* OR follow-up OR risk* OR prevalence OR longitudinal OR mortality OR retrospective OR “life course” OR transversal OR future OR cohort OR prospective).ti,ab,kf. OR associat*.ti. | exp Risk Factors/ OR Cross-Sectional Studies/ OR cohort studies/ OR longitudinal studies/ OR follow-up studies/ OR prospective studies/ OR retrospective studies/ OR Prevalence/ |
| Reviews | "systematic review".pt. or "Systematic Reviews as Topic"/ or "Cochrane Database of Systematic Reviews".jn. or (((comprehensive* or integrative or mapping or rapid or realist or scoping or systematic or systematical or systematically or systematicaly or systematicly or umbrella) adj3 (bibliographical or bibliographically or bibliographics or literature or review or reviews)) or (state adj3 art adj1 review) or (research adj2 synthesis) or ((data or information) adj3 synthesis)).ti,ab,kf. or ((data adj2 (extract or extracting or extractings or extraction or extraction)).ti,ab,kf. and ("review".ti. or "review".pt.)) or (((electronic or searched) adj2 database*) and (eligibility or excluded or exclusion or included or inclusion)).ti,ab,kf. or (overview adj4 reviews).ti,ab,kf. or  ((review adj3 (rationale or evidence)).ti,ab. and "review".pt.) or (PRISMA or (preferred adj1 reporting)).ab. or (cinahl or (cochrane adj3 (trial or trials)) or embase or medline or psyclit or (psycinfo not (psycinfo adj1 database)) or pubmed or scopus or (sociological adj1 abstracts) or (web adj2 science)).ab. | |

**Kidney Ovid MEDLINE – Strategy 2**

(preterm* or pre-matur* or preterm* or pre term? or “birth weight”).ti,kf. AND (kidney* or renal* or or nephr*).ti,kf.

**Hypertension Ovid MEDLINE – Strategy 1**

| **Concept** | **Keywords** | **MeSH** |
| --- | --- | --- |
| Preterm Neonate | (preterm or pre-mature or pre-matures or preterms or prematurity or pre-maturity or preterm or preterms or pre term? or preemie or preemies or premies or premie or  VLBW or VLBWI or VLBW-I or VLBWs or LBW or LBWI or LBWs or ELBW or ELBWI or ELBWs).ti,ab,kf. | exp Infant, Preterm/  exp Infant, Low Birth Weight/  exp Preterm Birth/ |
| Hypertension | (hypertens* or “high blood pressure”)ti,ab,kf. | Hypertension/ |
| Follow up/Outcomes | (outcome* OR follow-up OR risk* OR prevalence OR longitudinal OR mortality OR retrospective OR “life course” OR transversal OR future OR cohort OR cross-sectional OR prospective).ti,ab,kf. OR associat*.ti. | exp Risk Factors/ OR cohort studies/ OR Cross-Sectional Studies/ OR longitudinal studies/ OR follow-up studies/ OR prospective studies/ OR retrospective studies/ OR Prevalence/ |
| Reviews | As above |  |

**Kidney Embase – Strategy 1**

| **Concept** | **Keywords** | **Emtree** |
| --- | --- | --- |
| Preterm Neonate | preterm:ti,ab,kw OR 'pre mature':ti,ab,kw preterm:ti,kw OR 'pre mature':ti,kw OR 'pre matures':ti,kw OR preterms:ti,kw OR preterm:ti,ab,kw OR preterms:ti,ab,kw OR 'pre term':ti,ab,kw OR preemie:ti,ab,kw OR preemies:ti,ab,kw OR premies:ti,ab,kw OR premie:ti,ab,kw OR vlbw:ti,ab,kw OR vlbwi:ti,ab,kw OR 'vlbw i':ti,ab,kw OR vlbws:ti,ab,kw OR lbw:ti,ab,kw OR lbwi:ti,ab,kw OR lbws:ti,ab,kw OR elbw:ti,ab,kw OR elbwi:ti,ab,kw OR elbws:ti,ab,kw | 'prematurity'/exp OR 'low birth weight'/de OR 'very low birth weight'/de |
| Kidney outcomes (e.g. kidney size, kidney function by glomerular filtration rate, tubular function, or blood pressure) | kidney*:ti,kw OR renal*:ti,kw OR intrarenal:ti,kw OR nephr*:ti,kw OR glomerul*:ti,kw OR tubulopath*:ti,kw OR 'tubular function':ti,kw | 'kidney function'/exp/mj OR 'kidney disease'/exp/mj OR 'kidney weight'/exp/mj OR 'kidney size'/exp/mj OR 'kidney tumor'/exp/mj OR 'kidney function test'/exp/mj OR 'renal replacement therapy'/exp/mj OR 'kidney circulation'/exp/mj OR 'glomerulus filtration rate'/exp/mj |
| Follow up/Outcomes | outcome*:ti,ab,kw OR 'follow up':ti,ab,kw OR risk*:ti,ab,kw OR prevalence:ti,ab,kw OR longitudinal:ti,ab,kw OR mortality:ti,ab,kw OR retrospective:ti,ab,kw OR 'life course':ti,ab,kw OR transversal:ti,ab,kw OR cohort:ti,ab,kw OR 'cross sectional':ti,ab,kw OR prospective:ti,ab,kw OR associat*:ti | 'prospective study'/de OR 'cohort analysis'/de OR 'retrospective study'/de OR 'follow up'/de OR 'longitudinal study'/de OR 'prevalence'/de OR 'risk factor'/de |
| Reviews | 'systematic review'/de OR 'systematic review (topic)'/de OR (('comprehensive':ti,ab,kw OR 'integrated':ti,ab,kw OR 'integrative':ti,ab,kw OR 'mapping':ti,ab,kw OR 'methodology':ti,ab,kw OR 'narrative':ti,ab,kw OR 'scoping':ti,ab,kw OR 'systematic':ti,ab,kw) AND ('search':ti,ab,kw OR 'searched':ti,ab,kw OR 'searches':ti,ab,kw OR 'studies':ti,ab,kw) AND ('cinahl':ti,ab,kw OR 'cochrane':ti,ab,kw OR 'embase':ti,ab,kw OR 'psycinfo':ti,ab,kw OR 'pubmed':ti,ab,kw OR 'medline':ti,ab,kw OR 'scopus':ti,ab,kw OR 'web of science':ti,ab,kw OR 'bibliographic review':ti,ab,kw OR 'bibliographic reviews':ti,ab,kw OR 'literature review':ti,ab,kw OR 'literature reviews':ti,ab,kw OR 'literature search':ti,ab,kw OR 'literature searches':ti,ab,kw OR 'narrative review':ti,ab,kw OR 'narrative reviews':ti,ab,kw OR 'qualitative review':ti,ab,kw OR 'qualitative reviews':ti,ab,kw OR 'quantitative review':ti,ab,kw OR 'quantitative reviews':ti,ab,kw)) OR 'comprehensive review':ti,ab,kw OR 'comprehensive reviews':ti,ab,kw OR 'comprehensive search':ti,ab,kw OR 'comprehensive searches':ti,ab,kw OR 'critical review':ti,ab,kw OR 'critical reviews':ti,ab,kw OR (('electronic database':ti,ab,kw OR 'electronic databases':ti,ab,kw OR databases NEAR/3 searched) AND  (eligibility:ti,ab,kw OR excluded:ti,ab,kw OR exclusion:ti,ab,kw OR included:ti,ab,kw OR inclusion:ti,ab,kw)) OR 'evidence assessment':ti,ab,kw OR 'evidence review':ti,ab,kw OR 'exploratory review':ti,ab,kw OR 'framework synthesis':ti,ab,kw OR 'Integrated review':ti,ab,kw OR 'integrated reviews':ti,ab,kw OR 'integrative review':ti,ab,kw OR 'integrative reviews':ti,ab,kw OR 'mapping review':ti,ab,kw OR 'meta-review':ti,ab,kw OR 'meta-synthesis':ti,ab,kw OR 'methodology review':ti,ab,kw OR 'mixed methods review':ti,ab,kw OR 'mixed methods synthesis':ti,ab,kw OR (overview NEAR/4 reviews) OR 'PRISMA':ab OR ('preferred':ti,ab,kw and reporting:ti,ab,kw) OR 'prognostic review':ti,ab,kw OR 'psychometric review':ti,ab,kw OR 'rapid evidence assessment':ti,ab,kw OR 'rapid literature review':ti,ab,kw OR 'rapid literature search':ti,ab,kw OR 'rapid realist':ti,ab,kw OR 'rapid review':ti,ab,kw OR 'rapid reviews':ti,ab,kw OR 'realist review':ti,ab,kw OR 'review of reviews':ti,ab,kw OR 'scoping review':ti,ab,kw OR 'scoping reviews':ti,ab,kw OR 'scoping study':ti,ab,kw OR 'state of the art review':ti,ab,kw OR 'systematic evidence map':ti,ab,kw OR 'systematic evidence mapping':ti,ab,kw OR 'systematic literature':ti,ab,kw OR 'systematic Medline':ti,ab,kw OR 'systematic PubMed':ti,ab,kw OR 'systematic review':ti,ab,kw OR 'systematic reviews':ti,ab,kw OR 'systematic search':ti,ab,kw OR 'systematic searches':ti,ab,kw OR 'systematical literature review':ti,ab,kw OR 'systematical review':ti,ab,kw OR 'systematical reviews':ti,ab,kw OR 'systematically identified':ti,ab,kw OR 'systematically review':ti,ab,kw OR 'systematically reviewed':ti,ab,kw OR 'umbrella review':ti,ab,kw OR 'umbrella reviews':ti,ab,kw OR '13616137':is OR 'Cochrane Database of Systematic Reviews'/jt | |
| Human | #.. NOT ([animals]/lim NOT [humans]/lim) | |
| Not pregnancy | Not pregnancy or gestational hypertension | |
|  | Exclude Case Reports | |

**Kidney Embase – Strategy 2**

(preterm*:ti,kw OR 'pre matur*':ti,kw OR preterm*:ti,kw OR 'pre term':ti,kw OR 'birth weight':ti,kw) AND (kidney*:ti,kw OR renal*:ti,kw OR nephr*:ti,kw)

AND (study types above)

**Hypertension Embase – Strategy 1**

| **Concept** | **Keywords** | **Emtree** |
| --- | --- | --- |
| Preterm Neonate | preterm:ti,ab,kw OR 'pre mature':ti,ab,kw OR 'pre matures':ti,ab,kw OR preterms:ti,ab,kw OR prematurity:ti,ab,kw OR 'pre maturity':ti,ab,kw OR preterm:ti,ab,kw OR preterms:ti,ab,kw OR 'pre term':ti,ab,kw OR preemie:ti,ab,kw OR preemies:ti,ab,kw OR premies:ti,ab,kw OR premie:ti,ab,kw OR vlbw:ti,ab,kw OR vlbwi:ti,ab,kw OR 'vlbw i':ti,ab,kw OR vlbws:ti,ab,kw OR lbw:ti,ab,kw OR lbwi:ti,ab,kw OR lbws:ti,ab,kw OR elbw:ti,ab,kw OR elbwi:ti,ab,kw OR elbws:ti,ab,kw | 'prematurity'/exp OR 'low birth weight'/de OR 'very low birth weight'/de |
| Hypertension | hypertension:ti,kw OR 'high blood pressure':ti,kw | 'hypertension'/mj |
| Follow up/Outcomes | outcome*:ti,ab,kw OR 'follow up':ti,ab,kw OR risk*:ti,ab,kw OR prevalence:ti,ab,kw OR longitudinal:ti,ab,kw OR mortality:ti,ab,kw OR retrospective:ti,ab,kw OR 'life course':ti,ab,kw OR transversal:ti,ab,kw OR future:ti,ab,kw OR cohort:ti,ab,kw OR 'cross sectional':ti,ab,kw OR prospective:ti,ab,kw OR associat*:ti | 'prospective study'/de OR 'cohort analysis'/de OR 'retrospective study'/de OR 'follow up'/de OR 'longitudinal study'/de OR 'prevalence'/de OR 'risk factor'/de |
| Reviews | 'systematic review'/de OR 'systematic review (topic)'/de OR (('comprehensive':ti,ab,kw OR 'integrated':ti,ab,kw OR 'integrative':ti,ab,kw OR 'mapping':ti,ab,kw OR 'methodology':ti,ab,kw OR 'narrative':ti,ab,kw OR 'scoping':ti,ab,kw OR 'systematic':ti,ab,kw) AND ('search':ti,ab,kw OR 'searched':ti,ab,kw OR 'searches':ti,ab,kw OR 'studies':ti,ab,kw) AND ('cinahl':ti,ab,kw OR 'cochrane':ti,ab,kw OR 'embase':ti,ab,kw OR 'psycinfo':ti,ab,kw OR 'pubmed':ti,ab,kw OR 'medline':ti,ab,kw OR 'scopus':ti,ab,kw OR 'web of science':ti,ab,kw OR 'bibliographic review':ti,ab,kw OR 'bibliographic reviews':ti,ab,kw OR 'literature review':ti,ab,kw OR 'literature reviews':ti,ab,kw OR 'literature search':ti,ab,kw OR 'literature searches':ti,ab,kw OR 'narrative review':ti,ab,kw OR 'narrative reviews':ti,ab,kw OR 'qualitative review':ti,ab,kw OR 'qualitative reviews':ti,ab,kw OR 'quantitative review':ti,ab,kw OR 'quantitative reviews':ti,ab,kw)) OR 'comprehensive review':ti,ab,kw OR 'comprehensive reviews':ti,ab,kw OR 'comprehensive search':ti,ab,kw OR 'comprehensive searches':ti,ab,kw OR 'critical review':ti,ab,kw OR 'critical reviews':ti,ab,kw OR (('electronic database':ti,ab,kw OR 'electronic databases':ti,ab,kw OR databases NEAR/3 searched) AND  (eligibility:ti,ab,kw OR excluded:ti,ab,kw OR exclusion:ti,ab,kw OR included:ti,ab,kw OR inclusion:ti,ab,kw)) OR 'evidence assessment':ti,ab,kw OR 'evidence review':ti,ab,kw OR 'exploratory review':ti,ab,kw OR 'framework synthesis':ti,ab,kw OR 'Integrated review':ti,ab,kw OR 'integrated reviews':ti,ab,kw OR 'integrative review':ti,ab,kw OR 'integrative reviews':ti,ab,kw OR 'mapping review':ti,ab,kw OR 'meta-review':ti,ab,kw OR 'meta-synthesis':ti,ab,kw OR 'methodology review':ti,ab,kw OR 'mixed methods review':ti,ab,kw OR 'mixed methods synthesis':ti,ab,kw OR (overview NEAR/4 reviews) OR 'PRISMA':ab OR ('preferred':ti,ab,kw and reporting:ti,ab,kw) OR 'prognostic review':ti,ab,kw OR 'psychometric review':ti,ab,kw OR 'rapid evidence assessment':ti,ab,kw OR 'rapid literature review':ti,ab,kw OR 'rapid literature search':ti,ab,kw OR 'rapid realist':ti,ab,kw OR 'rapid review':ti,ab,kw OR 'rapid reviews':ti,ab,kw OR 'realist review':ti,ab,kw OR 'review of reviews':ti,ab,kw OR 'scoping review':ti,ab,kw OR 'scoping reviews':ti,ab,kw OR 'scoping study':ti,ab,kw OR 'state of the art review':ti,ab,kw OR 'systematic evidence map':ti,ab,kw OR 'systematic evidence mapping':ti,ab,kw OR 'systematic literature':ti,ab,kw OR 'systematic Medline':ti,ab,kw OR 'systematic PubMed':ti,ab,kw OR 'systematic review':ti,ab,kw OR 'systematic reviews':ti,ab,kw OR 'systematic search':ti,ab,kw OR 'systematic searches':ti,ab,kw OR 'systematical literature review':ti,ab,kw OR 'systematical review':ti,ab,kw OR 'systematical reviews':ti,ab,kw OR 'systematically identified':ti,ab,kw OR 'systematically review':ti,ab,kw OR 'systematically reviewed':ti,ab,kw OR 'umbrella review':ti,ab,kw OR 'umbrella reviews':ti,ab,kw OR '13616137':is OR 'Cochrane Database of Systematic Reviews'/jt | |
| Human | #.. NOT ([animals]/lim NOT [humans]/lim) | |

**Hypertension Embase – Strategy 2**

(preterm*:ti,ab,kw OR 'pre matur*':ti,ab,kw OR preterm*:ti,ab,kw OR 'pre term':ti,ab,kw OR 'birth weight':ti,ab,kw) AND (hypertension*:ti,kw OR ‘high blood pressure’:ti,kw)

(study types above)
